# Improved Function With Enhanced Protein Intake per Meal: A Pilot Study of Weight Reduction in Frail, Obese Older Adults

**DOI:** 10.1093/gerona/glv210

**Published:** 2016-01-18

**Authors:** Kathryn N. Porter Starr, Carl F. Pieper, Melissa C. Orenduff, Shelley R. McDonald, Luisa B. McClure, Run Zhou, Martha E. Payne, Connie W. Bales

**Affiliations:** ^1^ Center for the Study of Aging, Duke University Medical Center, Durham, North Carolina.; ^2^ Geriatric Research, Education, and Clinical Center, Durham VA Medical Center, North Carolina.; ^3^ Department of Biostatistics and Bioinformatics,; ^4^ Department of Medicine, and; ^5^ Department of Psychiatry and Behavioral Sciences, Duke University Medical Center, Durham, North Carolina.

**Keywords:** Obesity, Function, Frailty, Older adults, Weight loss intervention, Protein

## Abstract

**Background:**

Obesity is a significant cause of functional limitations in older adults; yet, concerns that weight reduction could diminish muscle along with fat mass have impeded progress toward an intervention. Meal-based enhancement of protein intake could protect function and/or lean mass but has not been studied during geriatric obesity reduction.

**Methods:**

In this 6-month randomized controlled trial, 67 obese (body mass index ≥30kg/m^2^) older (≥60 years) adults with a Short Physical Performance Battery score of 4–10 were randomly assigned to a traditional (Control) weight loss regimen or one with higher protein intake (>30g) at each meal (Protein). All participants were prescribed a hypo-caloric diet, and weighed and provided dietary guidance weekly. Physical function (Short Physical Performance Battery) and lean mass (BOD POD), along with secondary measures, were assessed at 0, 3, and 6 months.

**Results:**

At the 6-month endpoint, there was significant (*p* < .001) weight loss in both the Control (−7.5±6.2kg) and Protein (−8.7±7.4kg) groups. Both groups also improved function but the increase in the Protein (+2.4±1.7 units; *p* < .001) was greater than in the Control (+0.9±1.7 units; *p* < .01) group (*p* = .02).

**Conclusion:**

Obese, functionally limited older adults undergoing a 6-month weight loss intervention with a meal-based enhancement of protein quantity and quality lost similar amounts of weight but had greater functional improvements relative to the Control group. If confirmed, this dietary approach could have important implications for improving the functional status of this vulnerable population (ClinicalTrials.gov identifier: NCT01715753).

The obesity epidemic is progressively threatening the health of many older adults (1); already, 35% or more of those older than 60 years are obese (body mass index ≥30kg/m^2^) and this prevalence is expected to increase (2). While obesity is well known to intensify age-related chronic health conditions like cardiovascular disease and type 2 diabetes, its impact on function is even more pervasive. Essentially all older adults who are obese have diminished mobility, independence, and performance of activities of daily living (3,4). Excess adiposity, physical inactivity, and loss of muscle strength and mass are mutually and dramatically reinforcing, especially in combination with aging (4). Concerns are building that, in the near future, the functionally disabled, obese older adult may become the most commonly encountered phenotype of frailty (5).

Because of concerns about detrimental effects on muscle (6), bone (7), and/or mortality (8), weight loss has not been recommended for obese older adults. However, obesity reduction can provide remarkable benefits to inflammatory status, metabolic profiles, physical function, and quality of life in this population (1,9,10). Most geriatric obesity reduction trials to date have employed a diet plus exercise combination, as exercise drives muscle anabolism and can help preserve lean mass (LM) during weight loss (11,12). However, a subset of obese older adults are limited by excess weight and reduced muscle strength, making vigorous exercise an impractical option and limiting generalizability of previous findings to a frail, obese population (13,14). For those individuals with limited ability to exercise, there is an intriguing possibility that enhanced protein intake alone can be used to promote muscle protein synthesis and, thus, retention of LM, during obesity reduction. While the anabolic response to dietary protein is blunted by aging (14), enhancing the diet to include approximately 30g of high-quality protein per meal can enable older adults to reach a level of muscle protein synthesis equal to that of younger individuals (15–17). There is a growing consensus based upon these short-term studies that this level of protein per meal be recommended for older adults at risk for sarcopenia (17–20). This would represent a major shift in protein intake patterns, because the majority of adults skew their meal intake so that most of the protein for the day is consumed at the evening meal (17).

Whether per meal enhancement of protein intake can offset the lack of exercise and protect muscle mass and function during weight loss in frail but obese older adults is essentially unknown. In the only investigation to date, Coker and colleagues (13) compared a conventional meal replacement to one with whey protein and essential amino acids in a short-term (8 week) study. They found no group difference in LM preservation; however, the supplemented group had increased rates of muscle protein synthesis and preferential loss of adipose tissue. Based on this and the acute studies of protein synthesis thresholds for older adults (15,16), we developed the MEASUR-UP (Measuring Eating, Activity and Strength: Understanding the Response-Using Protein) trial, which is the first to examine meal-based enhancement of protein intake (30g high-quality protein per each of 3 meals) during a long-term (6 month) weight loss intervention (10% weight reduction goal) in obese older adults with functional limitations. Because animal proteins, complete in all essential amino acids, are superior for promoting anabolism (21) and considering the protein sources used in the short-term studies of protein enhancement (Symons et al. (15)—beef; Pennings et al. (16)—whey), the 30g of protein was provided from animal sources, primarily lean beef, and from other lean meats, low fat dairy, fish, and eggs. We hypothesized that participants following the enhanced protein regimen would (i) have greater improvements in functional status and (ii) have better preservation of LM than Control participants.

## Methods

### Trial Design and Randomization

**Figure 1.**
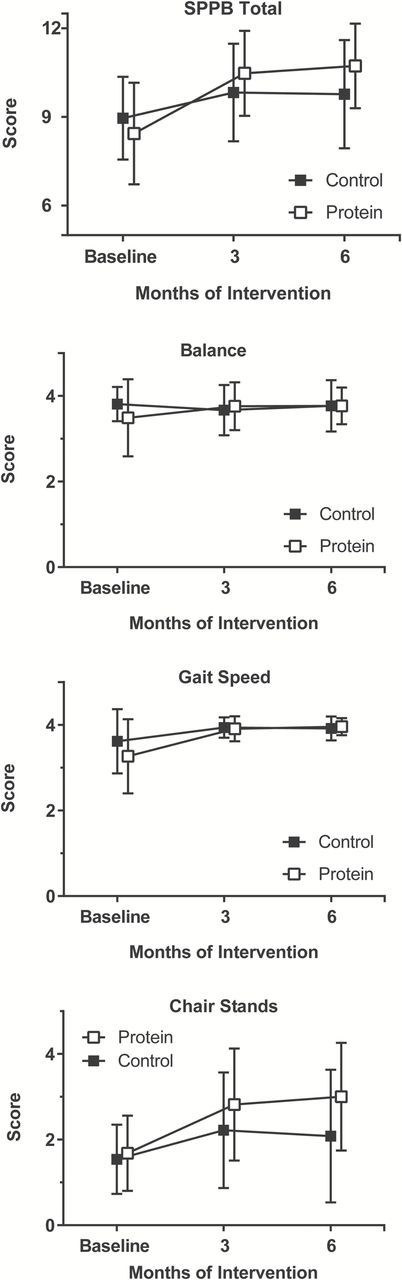
Mean changes in SPPB total score, balance, gait speed, and chair stands by group at 3 and 6 months of intervention.

MEASUR-UP is a two-armed randomized, controlled pilot trial, with primary and secondary outcomes assessed at 0, 3, and 6 months; the design and detailed methods were previously published (22). Blocking by gender and marital and partner status, eligible participants were randomly assigned to a traditional weight loss (Control) or a protein-enhanced weight loss (Protein) study arm in a 1:2 allocation ratio using a computerized randomization scheme. Primary outcomes were function (Short Physical Performance Battery; SPPB) (23) and LM (BOD POD). Secondary outcomes included anthropometrics, physical activity, and hand grip strength. Protein and calorie (kcal) intakes were assessed as indicators of feasibility and compliance. The study was approved by the Duke University Health System Institutional Review Board and written informed consent was obtained from all participants (Figure 1).

### Participants

Obese (body mass index ≥30kg/m^2^), functionally impaired (SPPB score of 4–10 out of 12), men and women living in communities near Durham, North Carolina, were recruited. The enrollment age of ≥60 years is a commonly used criteria for studies of older adults and was chosen because age-associated disabilities often appear earlier in individuals who are overweight and obese. All participants with a glomerular filtration rate (GFR) of ≥60mL/min/1.73 m^2^ were study eligible; those with a GFR of 45 to 59mL/min/1.73 m^2^ were enrolled but a GFR was repeated every 2 months. Exclusion criteria included GFR <45mL/min/1.73 m^2^, dementia, neurological conditions causing functional limitations, and unstable or terminal medical conditions.

### Interventions

All participants received a supervised weight loss treatment (hypo-caloric diet with goal of 10% weight loss over 6 months) delivered by Registered Dietitians (Interventionists). Interventionists provided individualized kcal prescriptions and meal plans, led weekly group meetings for counseling and peer support, and supervised weekly weigh-ins. A daily low dose multivitamin (Teen Multivitamin for Boys 12–17, GNC Milestones), along with calcium (400mg) and vitamin D (600 IU) (Citracal, Bayer), were provided to participants in both arms; other dietary supplements were prohibited during the trial. Adherence was assessed by 3-day food records and food journals (turned in at weekly group meetings), weight loss, and weekly attendance at group meetings. There was no prescribed exercise intervention; however, participants were encouraged to be as active as they safely could.

#### Diets

Control participants were prescribed a 500 kcal deficit diet (15% protein, 30% fat, 55% carbohydrate); prescribed protein intake met the Recommended Dietary Allowance (0.8g/kg body weight). Protein participants were also prescribed a 500 kcal deficit but with a macronutrient distribution of 30% protein, 30% fat, 40% carbohydrate; prescribed protein intake was 1.2g/kg. Protein meal plans included 30+ grams of lean, high-quality protein three times a day, with no adjustment for changes in body weight over time. To ease the economic and logistical burden, Protein participants were supplied with ≥420g protein per week (at least 60g per day, enough for two meals for each of the 7 days); it was provided in the form of cooked, then chilled or frozen, very lean beef (ground sirloin, deli roast beef, and flank steak), along with instructions for preparation. The preportioned lean beef was packed in insulated thermal bags and distributed at weekly group meetings. Other complete proteins (eg, other lean meats and poultry, low fat dairy foods and eggs) were consumed at the remaining meal (participant’s choice). This was to avoid monotony and to allow flexibility (eg, special occasions). Interventionists reviewed participants’ daily food journals each week and adjusted their menus to ensure the target of 30g protein per meal for breakfast, lunch, and dinner was met, as previously described (22). Weekly body weights provided an additional indication of diet compliance.

### Outcome Measurements

Physical function was measured using the SPPB (23), which has three component domains (balance, lower-body strength, and gait speed), yielding a total score of 0 (poor) to 12 (high). LM and fat mass were measured using BOD POD air displacement plethysmography (Life Measurement Inc, Concord, CA); the BOD POD was calibrated daily using the precision 49.367-L cylinder provided by the instrument manufacturer and measurement conditions (fasting state, clothing) were carefully standardized. Waist circumference (minimal waist, Gulick II tape measure) was also assessed. Isometric grip strength was assessed (both hands, Jamar Hand Dynamometer), with the higher of two successive scores for the dominant hand being recorded. Duration of moderate–intensity activities was assessed using the Community Healthy Activities Model Program for Seniors (CHAMPS) questionnaire (24).

For diet compliance, 3-day food records were collected at 0, 3, and 6 months and analyzed (Food Processor Nutrition Analysis Software; ESHA Research, Salem, OR) for daily intakes of kcals and protein, and protein and meal. Most study outcomes were objectively measured (eg, BOD POD output, distance walked in 6 minutes, self-administered questionnaires, etc.), and data management and analysis were performed by research staff fully blinded to treatment assignment. For the few potentially subjective measures, for example, coding of food choices from food records, the analysis was performed by staff blinded to treatments.

### Safety

Adverse events were monitored throughout the trial. Renal function (GFR; LabCorp, Inc) was assessed at baseline, every other month for those with a baseline GFR of 45 to <60mL/min/1.73 m^2^, and at the 6-month endpoint.

### Data Management and Analysis

The MEASUR-UP trial evaluated the efficacy of meal-based enhancement of protein during weight loss by comparing the Protein treatment to the Control. The design included an interim measurement point (3 months) to provide a more precise assessment of the functional form of the compliance by change in the two primary outcomes of function (SPPB) and lean body mass. The study analysis was performed under an “intent to treat” criteria, including all participants, whether compliant to the intervention or not. Data were double entered with differences adjudicated. Treatment codes were revealed only after locking of the database at trial’s end.

We tested primary and secondary variables with regards to overall change within arms and differences in change between arms over time. A Mixed Models repeated measures approach was used to assess change from baseline at two time points (3 and 6 months), controlling for baseline values. The main effect of the primary outcomes was tested by the time effect, while the group difference was assessed by statistical significance of the Group and the Group × Time interaction. Statistical significance was declared at level alpha of 0.05 (two-tailed*).* Meal enhancement of protein intake has not been previously studied during weight reduction in obese older adults with regards to our study outcomes, thus a priori power calculations were not possible. However, we carried out a post hoc power analysis using the derived parameters from the current trial to determine the necessary sample size. For SSPB and LM, setting alpha to 0.05 (two-tailed), power to 80% and assuming our means and variances are replicated, we calculated a requirement of *n* = 48 and *n* = 336 analyzable participants for SPPB and LM, respectively. These calculations are based on LM measurements using the BOD POD; if, as, some studies suggest (25), measurements made with BOD POD have higher variability than those made with dual energy X-ray absorptiometry, our post hoc power numbers may overestimate the required pool size if X-ray absorptiometry was used for LM assessment.

## Results

### Study Population and Retention

Of 93 eligible individuals, 26 were excluded at screening (see Consort diagram in Supplementary Material). Baseline characteristics for the 67 participants enrolled (Control, *n* = 26 and Protein, *n* = 41) are shown in Table 1. Due to time constraints that limited the trial duration, we randomized 11 participants to complete a 3 rather than a 6-month intervention (4 to Protein, 7 to Control). Two of those randomized to Protein and four of the Control participants, respectively, completed the 3 months and were assessed for all outcomes.

**Table 1.** Descriptive Characteristics of Participants (Control *n* = 26, Protein *n* = 41; represented as mean ± *SD* or *n* [%])

	Control	Protein	Total
Age (y)	68.7±6.2	67.9±5.3	68.2±5.6
Body weight (kg)	101.8±17.1	104.1±21.1	103.2±19.6
BMI (kg/m^2^)	36.4±5.5	37.2±6.8	36.9±6.3
Gender			
Female (%)	20 (77)	33 (80)	53 (79)
Male (%)	6 (23)	8 (20)	14 (21)
Race			
Black (%)	4 (15)	13 (32)	17 (25)
White (%)	21 (81)	26 (63)	47 (70)
Education			
≤ High school (%)	6 (23)	9 (22)	15 (22)
> High school (%)	20 (77)	32 (78)	52 (78)
Energy intake (kcal)	1,880±416	1,985±792	1,947±680
Protein intake (g)	81.5±16.9	88.0±26.6	85.7±23.6
GFR (mL/min/1.73 m^2^)^*,†^	76.3±16.8	78.1±14.9	77.4±15.6

*Note*: BMI = body mass index; GFR = glomerular filtration rate.

*GFR was calculated as (mL/min/1.73 m^2^) = 175 × (Scr) − 1.154×(Age) − 0.203 × (0.742 female) × (1.212 Black).

^†^Normal GFR values were defined as ≥60mL/min/1.73 m^2^.

The study population was largely female (79.1%) and white (70.1%), with Class II obesity (mean body mass index = 37.1kg/m^2^). Dropout rates are shown in the consort diagram (Supplementary Material); we found no group differences in dropout rates or in predictors of dropout. The only predictor of dropping out was having a lower mean waist circumference (*p* < .05); it was 112.9±12.8cm for dropouts, compared with 120.6±12.9cm for study completers.

### Intervention Delivery and Adherence

By the 6-month endpoint, both groups had reduced their kcal intakes (Table 2) and achieved significant (*p* < .001) amounts of weight loss (Control −7.5±6.2kg; Protein −8.7±7.4kg) (Supplementary Material). Study completers had excellent attendance at weekly group and weigh-in meetings (Control = 87% ± 11%; Protein = 85% ± 10%). Analyses of 3-day diet records (at 3 and 6 months, compared with baseline) revealed excellent congruence with the prescribed protein intakes in the Protein group at all three meals; at 3 and 6 months respectively, protein intakes were 27.7±28.4g and 27.9±29.1g for breakfast; 36.3±17.6g and 36.3±15.9g for lunch; and 42.9±23.2g and 37.5±16.8g for dinner. In the Protein group, protein intake was 1.2g/kg/day at both time points. In contrast, protein intake in the Control group remained at 0.8g/kg/day at 3 and 6 months.

**Table 2.** Baseline and Change Scores at 3 and 6 Months for Body Weight and Dietary Intakes (Control *n* = 26, Protein *n* = 41; represented as mean ± *SD*)

Outcome	Baseline		3 mo			6 mo		
	Control	Protein	Control	Protein	*p* Value	Control	Protein	*p* Value
Body weight (kg)	101.8±17.1	104.1±21.1	−4.6±4.6*	−5.9±4.3*	.42	−7.5±6.2*	−8.7±7.4*	.48
Body weight (%)			−4.6±4.6*	−5.4±3.4*	.46	−7.5±6.4*	−8.1±6.8*	.52
Calorie intake (kcal)	1,880±416	1,985±792	−525±488*	−405±799*	.11	−422±437*	−496±809*	.49
Protein intake (g/d)	81.5±16.9	88.0±26.6	−8.5±20.0	28.3±38.3*	<.001	−10.8±19.7^†^	23.3±36.9*	<.001
Protein intake (% kcal)	18±5	19±6	4±6^†^	11±6*	<.001	2±3^‡^	11±6*	<.001
Carbohydrate intake (% kcal)	44±7	42±8	9±12*	−2±10	<.001	6±10^‡^	−4±13^‡^	.01
Fat intake (% kcal)	38±4	38±8	−10±9*	−8±8*	.31	−7±8^†^	−8±10*	.47
Protein intake breakfast (g)	12.2±7.5	17.4±7.2	1.2±6.0	10.0±11.6*	<.001	−1.2±9.5	11.0±13.2*	<.001
Protein intake lunch (g)	23.7±16.9	25.7±14.2	−6.8±18.7^‡^	11.4±17.2*	<.001	−10.1±14.2^‡^	10.6±15.2*	<.001
Protein intake dinner (g)	39.1±16.2	38.9±13.6	−4.0±10.1	3.9±22.5	.15	−4.2±19.9^†^	−1.5±16.4	.05
Protein intake (g/kg of body weight)	0.8±0.2	0.8±0.3	−0.02±0.2	0.3±0.4*	<.001	−0.03±0.2	0.3±0.4*	<.001

*Note: p* Values for the comparison within and between the groups of change from baseline to 3 and 6 months were calculated using the mixed model repeated measures analysis of variance (ANOVA) with baseline values as covariates.

*p < .001 for within group change from baseline to 3 and 6 mo.

^†^ *p* < .01 for within group change from baseline to 3 and 6 mo.

^‡^ *p* < .05 for within group change from baseline to 3 and 6 mo.

### Primary Outcomes

As shown in Table 3, both Control and Protein groups achieved substantial improvements in SPPB score, controlling for baseline (*p* < .01). However, the SPPB response in the Protein group was greater than in the Control group, as indicated by the overall group effect (*p* = .02). While Control SPPB score increased by 0.8±1.5 and 0.9±1.7 at 3 and 6 months (*p* < .01, both), the corresponding increases in Protein were 2.3±1.7 and 2.4±1.7 (*p* < .001, both). As is expected when body weight is reduced, LM decreased in both groups (Control −1.8±2.9kg; Protein −1.1±1.5kg; *p* < .01, both); there was no group difference in this change (*p* = .87 at 3 and *p* = .38 at 6 months) (Table 3).

**Table 3.** Baseline and Change Scores at 3 and 6 Months for Function and Body Composition by Treatment Group (Control *n* = 26, Protein *n* = 41; represented as mean ± *SD*)

	Baseline		3 mo			6 mo		
Outcome	Control	Protein	Control	Protein	*p* Value	Control	Protein	*p* Value
Primary outcome measures								
Lean body mass (kg)	53.9±9.6	52.8±10.3	−0.7±2.9	−0.8±1.4^||^	.87	−1.8±2.9^§^	−1.1±1.5^§^	.38
SPPB total score*	9.0±1.4	8.4±1.7	0.8±1.5^§^	2.3±1.7^‡^	.02	0.9±1.7^§^	2.4±1.7^‡^	.02
SPPB balance score*	3.8±0.4	3.5±0.9	−0.2±0.4	0.3±1.0	.31	0.0±0.4	0.3±0.8^||^	.80
SPPB gait speed score*	3.6±0.7	3.3±0.9	0.2±0.6‡	0.8±0.9^‡^	.57	0.3±0.7^‡^	0.7±0.9^‡^	.67
SPPB chair stands score*	1.5±0.8	1.7±0.9	0.8±1.3§	1.2±1.0^‡^	.13	0.6±1.6	1.5±1.1^‡^	.05
Secondary outcome measures								
Lean mass (%)			−1.7±5.9	−1.5±2.6^||^	.88	−3.8±6.1^§^	−2.1±3.0^§^	.37
Fat mass (kg)	48.2±13.2	51.3±15.8	−4.1±4.0^‡^	−5.1±4.1^‡^	.58	−6.0±5.6^‡^	−7.6±6.7^‡^	.39
Fat mass (%)			−1.6±2.8^§^	−2.2±2.2^‡^	.39	−2.2±3.6^§^	−3.4±3.1^‡^	.25
Waist circumference (cm)	106.3±11.1	107.2±11.7	−3.6±3.8^§^	−2.9±5.9^§^	.44	−3.9±5.7	−7.3±11.7^‡^	.50
Duration of moderate physical activity^†^ (min/wk)	272±330	224±247	−72±240	60±233	.14	221±324^§^	132±237^||^	.33
Handgrip strength (kg)	28.3±8.9	25.3±9.4	−0.5±3.1	0.5±4.1	.53	1.3±3.3	1.1±3.3	.69

*Note:* SPPB = Short Physical Performance Battery. *p* Values for the comparison within and between the groups of change from baseline to 3 and 6 months were calculated using the mixed model repeated measures analysis of variance (ANOVA) with baseline values as covariates.

*SPPB total score ranges from 0 to 12 and SPPB balance, gait speed, and chair stand scores from 0 to 4, with higher scores indicating better function.

^†^Duration of moderate physical activity was derived from Community Healthy Activities Model Program for Seniors (CHAMPS) questions 7, 9, 14–16, 19, 21, 23–26, 29–33, 37, 38, 40.

^‡^ *p* < .001 for within group change from baseline to 3 and 6 mo.

^§^ *p* < .01 for within group change from baseline to 3 and 6 mo.

^||^ *p* < .05 for within group change from baseline to 3 and 6 mo.

### Secondary Outcomes

Fat mass was reduced at 3 and 6 months in Control (−4.1±4.0 and -6.0±5.6kg; *p* < .001, *p* < .001) and Protein (−5.1±4.1 and −7.6±6.7kg; *p* < .001, *p* < .001) groups (Table 3), as was waist circumference (Control, *p* < .01, Protein, *p* < .01 at 3 months; 6 months, Protein only, *p* < .001). There was no group difference for change in either fat mass or waist circumference. Physical activity (CHAMPS questionnaire) was unchanged at 3 months (Control, *p* = .24, Protein, *p* = .34) but significantly increased at 6 months (Control, *p* < .01, Protein, *p* < .05), with no group difference at either time point. Handgrip strength remained unchanged throughout the trial for both groups.

### Safety

No adverse events related to the protocol and no serious adverse events occurred. One Protein participant was disqualified when his GFR dropped below 45mL/min/1.73 m^2^ and one Control participant who had remained qualified throughout the trial had a GFR of 42 at 6 months. Otherwise, no clinically important changes in GFR occurred.

## Discussion

The MEASUR-UP trial demonstrated that per meal protein enhancement of a weight reduction diet is feasible, safe, and more effective at improving physical function in obese and frail older adults than a traditional weight loss diet. Although the increase in overall dietary protein was modest (from 0.8 to 1.2g/kg body weight), improvements in function exceeded those seen in Controls.

### New Interest in Protein-Enhanced Weight Loss Regimens

Recent reports on nutrition in older adults have emphasized the potential value of protein supplements and/or protein-centric meals and the importance of complete (animal source) protein for maintaining muscle quality (26–28). An elevated anabolic threshold in aged muscle has also been confirmed (16). To date, three studies have explored protein supplementation during geriatric obesity reduction. Mojtahedi and colleagues (29) evaluated a whey protein supplement (25g twice daily for 6 months, plus exercise) and found no differential effects on physical function or LM. Likewise, in a small (*n* =11), short-term (8-weeks) study using whey protein plus essential amino acids and no exercise intervention, Coker and colleagues (13) found no protein effect on LM; functional outcomes were not reported. In contrast to these findings, Verreijen and colleagues (30) compared a combination supplement (daily 20.7g leucine-rich whey protein, vitamin D, other nutrients; 13 weeks) to an isocaloric control (with resistance training in both groups) and reported a protein benefit for appendicular muscle mass. This result could relate in part to resistance training, which is well known to additively enhance the effect of protein supplementation on anabolism (31). However, while function improved in this trial, there was no group difference.

In the MEASUR-UP trial, a protein benefit was clearly demonstrated for function, but we were unable to detect a link between change in function and change in LM at 3 (*r* = −0.23, *p* = .12) or 6 (*r* = 0.03, = 0.86) months. This finding is in contrast to the impressive body of evidence supporting a benefit of higher protein intakes to LM retention in the general adult population (32,33) and it could be that the global BOD POD assessment of LM was insufficiently sensitive (eg, as compared with X-ray absorptiometry measurement) to detect group differences in LM retention at the level of the skeletal muscle in our frail population. This notion is consistent with our post hoc power calculation indicating an insufficient sample size for detecting a change in LM. However, it is noteworthy that among MEASUR-UP participants who lost 5% or more of baseline body weight, the proportion of weight loss that was LM was 31.8% in the Control group compared with 18.4% in the Protein group. This difference might suggest that the enhanced protein diet had the hypothesized protective effect on LM, an effect that might be significant when tested with a larger sample and/or a more sensitive body composition methodology.

### Strengths and Limitations

As an intensive weight reduction trial in an understudied population, MEASUR-UP has several strengths. Successful recruitment demonstrated enthusiasm for weight loss interventions among the obese and physically frail older population and the feasibility of the weight loss protocol was also confirmed. The functional testing provided direct evidence of the meaningful benefits accrued when obesity is reduced. Also, the meal-based protein enhancement was feasible, as meal-based targets for protein and kcals were achieved. These findings, along with the absence of adverse side effects, support further study of the functional benefits that protein-enhanced meals might provide during weight reduction in obese older adults.

Limitations of MEASUR-UP include its modest sample size. Given the pilot nature of this study, we restricted our analyses to two primary outcome measures and did not correct for multiple testing. Additionally, the small control group and carry forward of data for the subjects who completed the 3-month only protocol may have caused artificial increases in variation. The dropout rates, while disappointing, were not unexpected given the intensive nature of the intervention and the frail population (see Supplementary Material). Most dropouts occurred because of changes in personal or health circumstances that were unrelated to the study. The provision of beef protein for 2 of 3 meals per day leads to limited generalizability, both in terms of LM effects and with regards to community application. However, with respect to LM, there is no prior evidence of a meaningful differential anabolic effect of one complete protein versus another. As to community applicability, our participants did not have trouble with adherence to the meal plans as the menus were simple and easy to follow. However, while the provision of beef products may have improved adherence, it is also possible that the ability to choose from a wider variety of complete protein foods may simplify diet delivery while maintaining the functional benefits.

## Conclusions

The MEASUR-UP trial demonstrated that a weight loss diet with enhanced meal-based protein intake was comparable to a traditional diet in achieving weight loss and superior to a traditional diet in terms of improving function. These results raise the possibility that protein-enhanced meals can foster optimization of dietary protein efficacy during weight reduction in obese and frail older adults. Future trials with larger sample sizes that include outcomes of function and LM retention, as well as community-based studies of feasibility, are well justified. Meal-based protein enhancement, combined with weight loss, has the potential to improve physical function in frail and obese older adults, improving their independence and lessening the risk of negative health outcomes in this high-risk population.

## Funding

This study was funding by the Beef Checkoff Program and received additional support from the National Institutes of Health (5T32 AG000029 to K.N.P.S.; BIRWCH K12 HD043446 to M.E.P.; 1P30 AG028716 to C.F.P.).

## Supplementary Material

Supplementary Data
