# Supplementary material for: Improved Function With Enhanced Protein Intake per Meal: A Pilot Study of Weight Reduction in Frail, Obese Older Adults
Source: J Gerontol A Biol Sci Med Sci. 2016 Jan 18;71(10):1369–75. doi: 10.1093/gerona/glv210 (PMC5018561; doi:10.1093/gerona/glv210)
Supplement: Supplementary Data [file glv210_supplementary_data.zip › Consort_Diagram_Supplementary.doc]

**Participant Screening, Enrolling, and Follow-up in MEASUR-UP Study**

**Allocation**

**Discontinued**

**Enrollment**

**150** individuals assessed for eligibility by phone screening

**57** Excluded

**17** Did not meet inclusion criteria

**11** BMI < 30

**3** Unable to attend intervention meetings

**2** Taking weight loss medication

**1** Age < 60

**24** Lost to contact

**3** Family emergency

**13** Other

  Other reasons (n= )

**9** Discontinued intervention

**9** Family/life obligations/complications

**30.7%** Dropout rate at 3-months

**5.3%** Dropout rate at 6-months

**26*** Randomized to Control group

**11** Discontinued intervention

**8** Family/life obligations/complications

**2** Illness

**1** GFR fell below 45 mL/min/1.73 m^2^

**17.1%** Dropout rate at 3-months

**10.8%** Dropout rate at 6-months

**41*** Randomized to Protein group

**67** Randomized

**93** Individuals signed a consent form

**26** Excluded

**7** Did not meet inclusion criteria

**5** Scored >10 on SPPB

**2** GFR below 45 mL/min/1.73 m^2^

**3** Too much of a commitment

**2** Schedule conflict with intervention

**14** Other

*Six participants were enrolled for 3 rather than 6-months due to time constraints on study completion

(Control n=4; Protein n=2).
